# Risk factors for surgical site infection and association of surgical site infection with survival of lower rectal cancer patients without clinical lateral pelvic lymph node metastasis (clinical Stage II/III): Analysis of data from JCOG0212

**DOI:** 10.1007/s10585-021-10117-8

**Published:** 2021-08-18

**Authors:** Kenji Katsumata, Masanobu Enomoto, Tetsuo Ishizaki, Shin Fujita, Yukihide Kanemitsu, Masaaki Ito, Akio Shiomi, Koji Komori, Masayuki Ohue, Mitsuyoshi Ota, Yoshihiro Akazai, Manabu Shiozawa, Takashi Yamaguchi, Hiroyuki Bando, Mitugu Sekimoto, Takaya Kobatake, Ryunosuke Machida, Takayuki Akasu, Yoshihiro Moriya, the Colorectal Cancer Study Group of Japan Clinical Oncology Group

**Affiliations:** 1 Department of Gastrointestinal and Pediatric Surgery, Tokyo Medical University, 6-7-1, Nishi-Shinjyuku Shinjyuku-ku, Tokyo, 160-0023 Japan; 2 Department of Surgery, Tochigi Cancer Center, Tochigi, Japan; 3 Department of Colorectal Surgery, National Cancer Center Hospital, Tokyo, Japan; 4 Department of Colorectal Surgery, National Cancer Center Hospital East, Chiba, Japan; 5 Division of Colon and Rectal Surgery, Shizuoka Cancer Center Hospital, Shizuoka, Japan; 6 Department of Surgery, Aichi Cancer Center Hospital, Nagoya, Japan; 7 Department of Gastroenterological Surgery, Osaka International Cancer Institute, Osaka, Japan; 8 Department of Surgery, Yokohama City University Medical Center, Kanagawa, Japan; 9 Department of Surgery, Okayama Saiseikai General Hospital, Okayama, Japan; 10 Department of Surgery, Kanagawa Cancer Center, Yokohama, Japan; 11 Department of Surgery, Kyoto Medical Center, Kyoto, Japan; 12 Department of Surgery, Ishikawa Prefectural Central Hospital, Ishikawa, Japan; 13 Department of Surgery, National Hospital Organization Osaka National Hospital, Osaka, Japan; 14 Department of Surgery, Shikoku Cancer Center, Ehime, Japan; 15 JCOG Data Center and Operations Office, National Cancer Center Hospital, Tokyo, Japan; 16 Department of Surgery, The Imperial Household Agency Hospital, Tokyo, Japan; 17 Department of Surgery, Japanese Red Cross Medical Center, Tokyo, Japan

**Keywords:** Rectal cancer, Latera lymph node dissection, Surgical site infection, Recurrence

## Abstract

This study aimed to examine the risk factors for surgical site infection (SSI) and the association of that with recurrence in JCOG0212. The results for secondary endpoints showed that compared with the mesorectal excision (ME) alone group, ME with lateral lymph node dissection (LLND) group showed significantly longer operative time and significantly higher blood loss. These results suggested that LLND was a risk factor for SSI. All 701 patients registered in JCOG0212 were analyzed in this study. Wound infection was defined as incisional/deep SSI, and pelvic abscess and anastomotic leakage were defined as organ/space SSI. The risk factors for the incidence of SSI and the effect of SSI on relapse-free survival (RFS) were investigated. Multivariable odds ratio of Grade 2 or higher all SSI was 0.58 [95% Confidence interval: 0.36–0.93] for female (vs. male) and that of Grade 2 or higher incisional/deep SSI was 2.24 [1.03–4.86] for blood infusion. For RFS, patients with Grade 3 or higher all SSI showed poor prognosis (multivariable hazard ratio: 1.66 [1.03–2.68]). LLND is not significant factor for the incidence of all SSI. Male sex might be a risk factor of Grade 2 or higher SSI, and blood transfusion is a possible risk factor of Grade 2 or higher incisional/deep SSI. Grade 3 or higher all SSI might be a significant worse prognostic factor for lower rectal cancer.

## Introduction

Surgical site infection (SSI), an infection associated with surgery, is a major complication. Generally, SSI is the second-most frequent occurring medical infection in developed countries [1]. One of the issues with the incidence of SSI with cancer surgery is that SSI leads to substantial health economic losses due to prolonged hospitalization [2]. Another issue is that the development of SSI is a poor prognostic factor of cancer [3]. Thus, countermeasures for SSI are important in medical economics and the prognosis of cancer. A wide range of evidence-based guidelines has been developed by the World Health Organization (WHO) [4]. and the Centers for Disease Control and Prevention [5]. In particular, the incidence of wound infection and anastomotic leakage is more frequent in rectal cancer than in other organ cancers [6]. Moreover, as mentioned above, many reports indicated that the development of SSI is a risk factor for cancer recurrence; this has also been suggested in colorectal cancer, indicating that this is an important issue [7].

In surgery for the treatment of lower rectal cancer, mesorectal excision (ME) with lateral pelvic lymph node dissection (LLND) is the standard surgical procedure for patients with advanced lower rectal cancer in Japan [8]. Autonomic nerve-sparing surgery with LLND was reportedly useful in low rectal cancer in many retrospective studies [9]. However, a randomized, controlled trial was essential to evaluate the benefits of extended lymphadenectomy for patients without extra-mesenteric metastasis. In order to acquire high-level evidence, the JCOG0212 randomized controlled trial was conducted in Japan.

On the other hand, ME with chemo-radiotherapy is the global standard treatment for rectal cancer. Therefore, we conducted a randomized controlled trial, JCOG0212, and concluded that the non-inferiority of ME alone could not be confirmed compared with ME with LLND [10].

To the best of our knowledge, JCOG0212 may be the only clinical trial worldwide that has been performed on LLND. By performing LLND, recurrences in the pelvis were reduced from 12.6 to 7.4%; however, the duration of surgery increased by 106 min, the volume of blood loss increased by 239 mL, and Grade 3 or higher complications were reported in 22% versus 16% of patients [11]. Generally, prolongation of the operative time and increase of the bleeding are major factors of the incidence of SSI. Even when these negative factors were considered, the results showed that LLND was the standard procedure performed. However, the issue of whether these negative aspects of LLND have any effect on SSI or on recurrence at the very least remained unresolved. Thus, in the present study, whether risk factors for the incidence of SSI and LLND were associated with SSI and whether SSI was a prognostic factor for relapse-free survival (RFS) were analyzed using data from the JCOG0212.

## Materials and methods

### Population

Inclusion criteria of JCOG0212 have been reported previously. Eligibility criteria included histologically proven rectal cancer at clinical Stage II or III; (T-factor T2–T3 and N factor regional lymph node except of pelvic lateral and root of inferior mesenteric arterial lymph node), with the lower margin being below the peritoneal reflection; performance status of 0 or 1; and age 20–75 years. A total of 701 patients were randomized to the ME with LLND group (n = 351) or the ME alone group (n = 350).

### Definitions of endpoints

Wound infection was defined as incisional/deep SSI, and pelvic abscess and anastomotic leakage were defined as organ/space SSI according to NCI-CTC ver.2.0 in the present study [12]. The definition of Grade is that Grade 0 is absent, Grade 1 is mild, Grade 2 is moderate, Grade 3 is severe, Grade 4 is life-threatening SSI. Grade 1 is local infection and does not affect a general condition. All SSI included incisional/deep SSI and organ/space SSI. The RFS and local RFS were same definitions in JCOG0212 [13].

### Statistical analysis

Each event numbers were small. Therefore risk factors for the incidence of Grade 2 or higher SSI were analyzed according to definitions of SSI (all SSI, incisional/deep SSI, and organ/space SSI). Odds ratio (OR) and its 95% confidence intervals (CI) for each factor were calculated by the multivariable logistic regression model in 691 patients without missing variables. The RFS and local RFS by definitions of SSI Grade were estimated according to the Kaplan–Meier method in all 701 patients. The effects of SSI on RFS and local RFS were evaluated by the multivariable Cox regression analysis in 693 patients without missing variables. The covariates were selected using a backward method with exclusion criteria of significance level of 0.20 under forcibly including the presence or absence of Grade 2 or higher and Grade 3 or higher SSI. Hazard ratio (HR) and its 95% CIs for each factor in the final model were described. A p-value of ≤ 0.05 was considered statistically significant. All statistical analyses were performed by SAS ver. 9.4.

## Results

### Risk factors for incidence of SSI

The grade 2 or higher all SSI was observed in 120 (17.1%) patients and all Grade 3 or higher in 48 (6.8%) patients (Table 1). The Grade 2 or higher incisional/deep SSI was observed in 55 (7.8%) patients and incisional/deep Grade 3 or higher SSI in 18 (2.6%) patients. Grade 2 or higher organ/space SSI was observed in 81 (11.6%) patients and Grade 3 or higher organ/space SSI in 32 (4.6%) patients.

**Table 1:** Incidence of Surgical site infection (SSI)

SSI	Grade 0	Grade 1	Grade 2	Grade 3	Grade 4	≥ Grade 2	≥ Grade 3	% ≥ Grade 2	% ≥ Grade 3	Total
Incisional/Deep	634	12	37	17	1	55	18	7.8	2.6	701
Organ/space	604	16	49	30	2	81	32	11.6	4.6	701
All (Incisional/Deep/Organ/space)	556	25	72	46	2	120	48	17.1	6.8	701

In the multivariable logistic regression, LLND was not associated with any SSI. In other factors, male sex was a significant risk factor for the incidence of all Grade 2 or higher SSI (OR: 0.58, 95% CI 0.36–0.93, p = 0.023). Presence of blood transfusions was a significant risk factor for the incidence of Grade 2 or higher incisional/deep SSI (OR: 2.24, 95% CI 1.03–4.86, p = 0.043). No significant factors for the incidence of Grade 2 or higher organ/space SSI were noted. (Table 2).

**Table 2:** Multivariable logistic regression analyses for Grade2 or higher SSI (n = 691)

SSI	Grade 0	Grade 1	Grade 2	Grade 3	Grade 4	≥ Grade 2	≥ Grade 3	% ≥ Grade 2	% ≥ Grade 3	Total
Incisional/Deep	634	12	37	17	1	55	18	7.8	2.6	701
Organ/space	604	16	49	30	2	81	32	11.6	4.6	701

Factors	Levels	All SSI			Incisional/Deep SSI			Organ/space SSI		
		Number of event/N	Odds ratio [95% CI]	p-value	Number of event/N	Odds ratio [95% CI]	p-value	Number of event/N	Odds ratio [95% CI]	p-value
Sex	Male	90/467	1	–	41/467	1	–	61/467	1	–
	Female	27/224	0.581 [0.364–0.927]	0.0227	12/224	0.634 [0.323–1.244]	0.1849	19/224	0.599 [0.347–1.034]	0.0660
Age	≤ 60	57/324	1	–	21/324	1	–	41/324	1	–
	> 60	60/367	0.845 [0.560–1.273]	0.4206	32/367	1.187 [0.657–2.144]	0.5704	39/367	0.812 [0.504–1.309]	0.3922
Comorbidities	Without	96/549	1	–	43/549	1	–	65/549	1	–
	With	21/142	0.822 [0.488–1.382]	0.4592	10/142	0.882 [0.427–1.824]	0.7350	15/142	0.866 [0.475–1.579]	0.6383
BMI	≤ 25	85/523	1	–	35/523	1	–	60/523	1	–
	> 25	32/168	1.193 [0.755–1.885]	0.4497	18/168	1.621 [0.881–2.984]	0.1204	20/168	1.055 [0.611–1.820]	0.8487
Operative time	≤ 4 h	23/151	1	–	10/151	1	–	19/151	1	–
	> 4 h	94/540	0.949 [0.539–1.672]	0.8570	43/540	0.899 [0.398–2.027]	0.7967	61/540	0.769 [0.406–1.455]	0.4195
Type of surgery	LAR	91/566	1	–	37/566	1	–	69/566	1	–
	APR	26/125	1.345 [0.802–2.254]	0.2612	16/125	1.814 [0.935–3.523]	0.0784	11/125	0.708 [0.353–1.418]	0.3301
LLND	Without	55/345	1	–	26/345	1	–	38/345	1	–
	With	62/346	1.122 [0.716–1.758]	0.6147	27/346	1.010 [0.536–1.901]	0.9762	42/346	1.203 [0.705–2.052]	0.4988
Tumor location	Ra	24/160	1	–	7/160	1	–	19/160	1	–
	Rb, P	93/531	1.113 [0.667–1.859]	0.6812	46/531	1.703 [0.728–3.984]	0.2195	61/531	1.059 [0.600–1.870]	0.8433
Blood transfusion	Without	100/623	1	–	43/623	1	–	70/623	1	–
	With	17/68	1.712 [0.930–3.152]	0.0844	10/68	2.237 [1.028–4.864]	0.0423	10/68	1.364 [0.653–2.851]	0.4092

Patients with missing data on BMI (n = 7) and type of surgery of Hartmann (n = 3) were excluded

*BMI* body mass index, *CI* confidence interval

### Associations of SSI with RFS and local RFS

The 5-year RFS for patients with Grade 1 or lower and Grade 2 or higher all SSI were 73.6% (95% CI 769.8–77.0%) and 72.5% (95% CI 63.5–79.6%), respectively (univariable HR: 0.942, 95% CI 0.65–1.33). For incisional/deep SSI, the 5-year RFS for patients with Grade 1 or lower and Grade 2 or higher SSI were 73.4% (95% CI 69.8–76.7%) and 72.6% (95% CI 58.8–82.5%), respectively (univariable HR: 0.906, 95% CI 0.55–1.51). For organ/space SSI, the 5-year RFS in patients with Grade 1 or lower and Grade 2 or higher SSI were 73.6% (95% CI 69.9–76.9%) and 71.6% (95% CI 60.4–80.1%), respectively (univariate HR: 0.995, 95% CI 0.65–1.52).

**Fig. 1:**
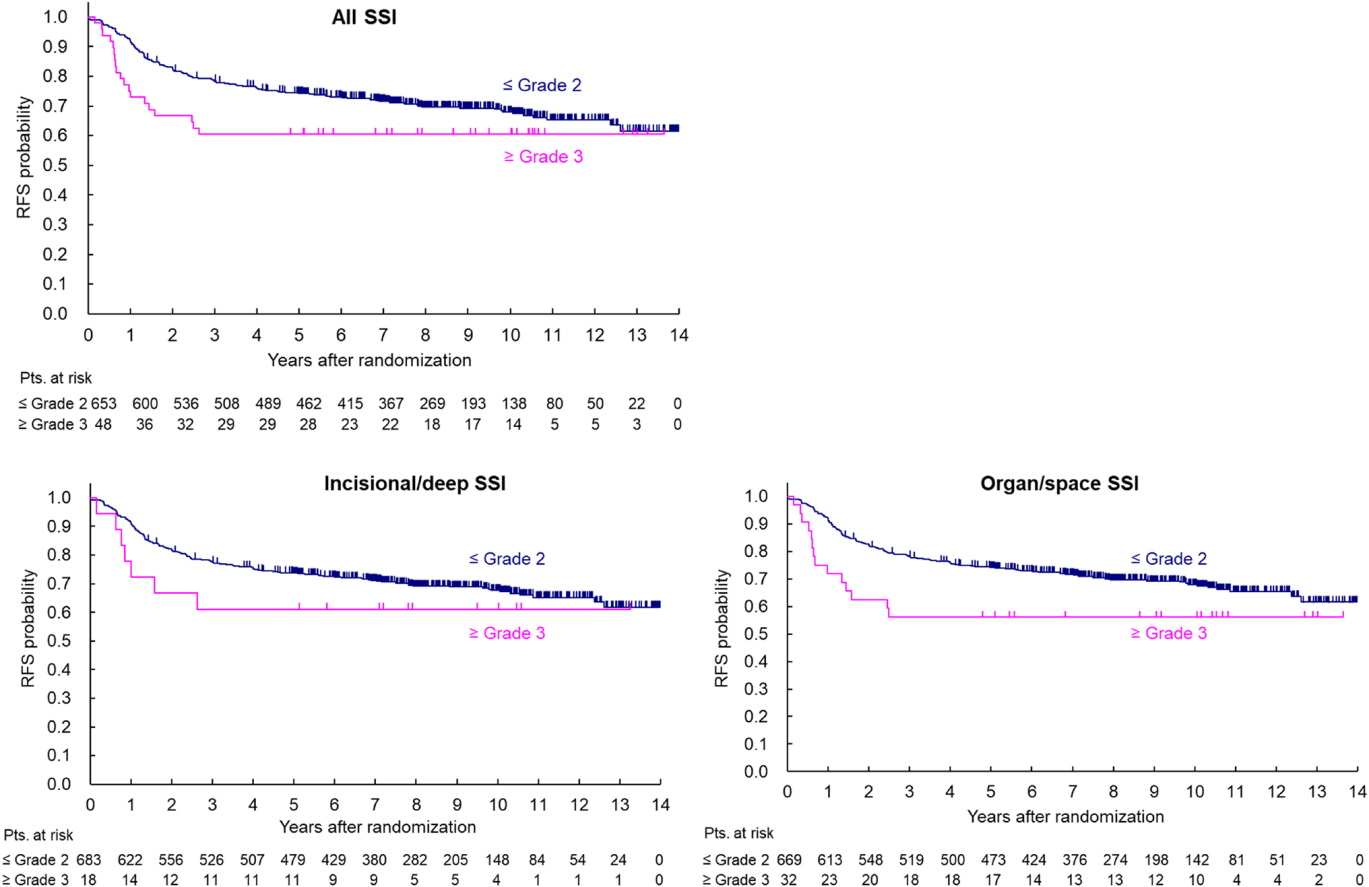
Relapse-free survival curves (Grade 2 or lower vs. Grade 3 or higher SSI); all SSI (top figure), incisional/deep SSI (bottom left), and organ/space SSI (bottom right)

The 5-year RFS for patients with Grade 2 or lower and Grade 3 or higher all SSI were 74.3% (95% CI 70.8–77.5%) and 60.4% (95% CI 45.2–72.6%), respectively (univariable HR: 1.49, 95% CI 0.93–2.39). For incisional/deep SSI, the 5-year RFS for patients with Grade 2 or lower and Grade 3 or higher SSI were 73.7% (95% CI 70.2–76.8%) and 61.1% (95% CI 35.3–79.2%), respectively (univariable HR: 1.45, 95% CI 0.68–3.08). For organ/space SSI, the 5-year RFS in patients with Grade 2 or lower and Grade 3 or higher SSI were 74.2% (95% CI 70.7–77.3%) and 56.3% (95% CI 37.6–71.3%), respectively (univariate HR: 1.71, 95% CI 0.99–2.93) (Fig. 1).

In the multivariable analysis for Grade 3 or higher all SSI, lymph node metastases, blood transfusions, pathological curative degree, pathological T stage, pathological N stage, and postoperative adjuvant chemotherapy were selected as the covariates in the final model RFS based on stepwise method. Regarding multivariable analyses for incisional/deep SSI and organ/space SSI, histological type for primary lesion was also selected as covariate. Patients with Grade 3 or higher all SSI showed poor RFS (HR: 1.66, 95% CI 1.03–2.68, p = 0.038). Incisional/deep Grade 3 or higher SSI was not a significant risk factor (HR: 1.43, 95% CI 0.66–3.13, p = 0.367), whereas organ/space SSI was significantly associated with RFS (HR: 2.01, 95% CI 1.16–3.49, p = 0.013). (Table 3). No significant differences were noted in the local RFS for any definitions of SSI. In addition, no large differences in presence or absence of LLND was observed.

**Table 3:** Multivariable Cox regression analyses for Grade 3 or higher SSI (n = 693)

		All SSI			Incisional/Deep SSI			Organ/space SSI		
Factors	Levels	Number of event/N	Hazard ratio [95% CI]	p-value	Number of event/N	Hazard ratio [95% CI]	p-value	Number of event/N	Hazard ratio [95% CI]	p-value
LLND	Without	111/345	1	–	111/345	1	–	111/345	1	–
	With	106/348	0.812 [0.618–1.068]	0.1367	106/348	0.827 [0.628–1.089]	0.1763	106/348	0.790 [0.599–1.043]	0.0964
Blood transfusion	Without	191/625	1	–	191/625	1	–	191/625	1	–
	With	26/68	1.538 [1.014–2.332]	0.0429	26/68	1.557 [1.020–2.377]	0.0401	26/68	1.649 [1.086–2.504]	0.0190
Histological type	Well/Mod				203/663	1	–	203/663	1	–
	Others				14/30	1.438 [0.827–2.501]	0.1985	14/30	1.482 [0.854–2.572]	0.1616
Curability	Cur A	205/679	1	–	205/679	1	–	205/679	1	–
	Cur B or C	12/14	3.908 [1.904–8.023]	0.0002	12/14	4.034 [1.946–8.361]	0.0002	12/14	4.456 [2.168–9.161]	< .0001
pT	≤ pT2	29/172	1	–	29/172	1	–	29/172	1	–
	≥ pT3	188/521	2.166 [1.452–3.230]	0.0002	188/521	2.137 [1.432–3.190]	0.0002	188/521	2.127 [1.426–3.173]	0.0002
pN	pN0	82/377	1	–	82/377	1	–	82/377	1	–
	pN +	135/316	3.373 [1.941–5.862]	< .0001	135/316	3.290 [1.888–5.731]	< .0001	135/316	3.159 [1.816–5.498]	< .0001
Adjuvant	Without	105/411	1	–	105/411	1	–	105/411	1	–
	with	112/282	0.585 [0.341–1.003]	0.0515	112/282	0.582 [0.340–0.999]	0.0495	112/282	0.618 [0.361–1.060]	0.0807
SSI grade	≤ 2	198/645	1	–	210/675	1	–	203/661	1	–
	≥ 3	19/48	1.660 [1.029–2.678]	0.0379	7/18	1.432 [0.657–3.125]	0.3667	14/32	2.009 [1.156–3.490]	0.0133

We included the following factors in the first models; sex (male or female), age (≤ 60 years old or ≥ 61 years old), operative time (≤ 4 h or > 4 h), type of surgery (LAP or APR), LLND (with or without), tumor location (Ra or Rb/P), blood transfusion (with or without), distances from anal verge (≤ 5 cm or > 5 cm), tumor size (≤ 5 cm or > 5 cm), histological type (well/mod or others), curability (cur A or cur B/C), pT (≤ pT2 or ≥ pT3), pN (pN0 or pN +), adjuvant (with or without), and each SSI grade (≤ 2 or ≥ 3), and the factors shown in table above were selected in the final models using backward elimination method under forcibly including SSI grade. Histological type was not selected in the model with all SSI grade. Patients with type of surgery of Hartmann (n = 3) and missing data on distances from anal verge (n = 5) were excluded

*CI* confidence interval

## Discussion

This study showed that male sex might be a risk factor of Grade2 or higher SSI, and blood transfusion is a possible risk factor of Grade 2 or higher incisional/deep SSI. Moreover, Grade 3 or higher SSI might be a significant prognostic factor for RFS in patients with lower rectal cancer. On the contrary, LLND was not associated with the incidence of any SSI, including incisional/deep and organ/space SSI.

The existing recommendation in guidelines such as those established by the WHO for preparation prior to colorectal cancer surgery is to use oral antibiotics and mechanical bowel preparation in conjunction with oral antibiotics the day prior to surgery [4]. At the time of JCOG0212, which was conducted from 2003 to 2010, it was presumed that many institutions used mechanical bowel preparation alone because intestinal pretreatment with a combination of oral antibiotics given daily for 3 days induced a high proportion of drug resistance. Kobayashi et al. reported that approximately 10% of patients received antibiotics the day prior to surgery in 2007 [14]. It is necessary to think about incidence of SSI of JCOG0212 in consideration of condition which was not good rather than present status.

Many factors related to SSI have been identified, such as smoking, hyperglycemia, anemia, and ASA score; in addition, male sex and colostomy have been suggested as factors related to SSI in rectal cancer [15]. In the present analyses, blood transfusions and male sex were found to be factors related to SSI. In contrast, for the involvement of LLND, blood transfusions tended to be significantly more prevalent in the TME and LLND groups. The incidence of Grade 3–4 postoperative complications was also higher [4]; however, to the best of our knowledge, in this study, it was demonstrated for the first time that LLND was not a risk factor for incidence of SSI in lower rectal surgery. The frequency of incidence of SSI in colorectal cancer surgery is higher than that in other gastrointestinal cancers, and many reports have been published on the association of cancer recurrence with SSI. Associations with recurrence [7] and those with local recurrence of rectal cancer [16], wound infection, anastomotic leakage, and all infections have been reported [17]. Although the results have not yet been confirmed, most reports indicate an association between recurrence and prognosis. There have been various reports on these mechanisms, and delays of adjuvant chemotherapy, systemic inflammation and cytokines, and implantation of cancer cells, among others [18], [19] have been observed.

This study showed an association between all and organ/space SSI and recurrence. Significant differences were recognized in patients with Grade 3 or higher SSI, and almost all patients experienced recurrence within 3 years. This suggested that in case of Grade 3 or higher SSI, patients have a systemic response that may affect cancer progression. This study suspected that Grade 3 or higher SSI induced poor prognosis in patients with systemic inflammatory responses, such as a high neutrophil–lymphocyte count ratio and high CRP, which has been shown in patients with advanced colorectal cancer [18]. However, it should be noted that Grade 3 or higher SSI were only reported in a limited number of patients (6.8% of the total) and this should be considered in the interpretation of the results.

Conversely, local RFS curves were similar and no effects were observed on local recurrence in this study. The incidence of SSI has negative effects on both medical economics and prognoses, and thus, sufficient countermeasures are necessary.

In addition, the 7-year overall survival (OS) for patients with Grade 2 or lower and Grade 3 or higher all SSI were 82.1% and 79.1%. For incisional/deep SSI, the 7-year OS for patients with Grade 2 or lower and Grade 3 or higher SSI were 82.4% and 77.8%. For organ/space SSI, the 7-year OS in patients with Grade 2 or lower and Grade 3 or higher SSI were 82.0% and 78.2%. Grade 3 or higher SSI did not affect the overall survival rate.

But this study has some limitations. One is that there were few events of SSI. Another one is that the primary endpoint of this clinical trial was not the association of SSI. And therefore we might not take enough dates for analysis.

In conclusion, the risk factors of the incidence of Grade 2 or higher all SSI was male sex and that of incisional/deep SSI was presence of blood transfusions. LLND was not associated with any SSI. The incidence of Grade 3 or higher all SSI and space/organ SSI was a significant prognostic factor in laparotomy for rectal cancer and requires preventive measures.

## Abbreviations

JCOG 0212: Mesorectal excision with or without lateral lymph node dissection for Clinical stage II/III lower rectal cancer, a multicenter, randomized controlled, noninferiority trial
CI: Confidence intervals
LLND: Lateral lymph node dissection
ME: Mesorectal excision
OR: Odds ratio
RFS: Recurrence-free survival
SSI: Surgical site infection
WHO: World Health Organization
